# Cocaine use and head and neck cancer risk: A pooled analysis in the International Head and Neck Cancer Epidemiology Consortium

**DOI:** 10.1002/cam4.7019

**Published:** 2024-02-24

**Authors:** Mingyan Zhang, Chu Chen, Guojun Li, Alzina Koric, Yuan‐Chin Amy Lee, Hal Morgenstern, Stephen M. Schwartz, Erich M. Sturgis, Paolo Boffetta, Mia Hashibe, Zuo‐Feng Zhang

**Affiliations:** ^1^ Department of Epidemiology UCLA Fielding School of Public Health Los Angeles California USA; ^2^ Program in Epidemiology, Division of Public Health Sciences Fred Hutchinson Cancer Center Seattle Washington USA; ^3^ Department of Head and Neck Surgery, Division of Surgery University of Texas M. D. Anderson Cancer Center Houston Texas USA; ^4^ Division of Public Health, Department of Family and Preventive Medicine University of Utah School of Medicine, and Huntsman Cancer Institute Salt Lake City Utah USA; ^5^ Departments of Epidemiology and Environmental Health Sciences, School of Public Health and Department of Urology, Medical School University of Michigan Ann Arbor Michigan USA; ^6^ Department of Otolaryngology‐Head and Neck Surgery Baylor College of Medicine Houston Texas USA; ^7^ Stony Brook Cancer Center, Department of Family, Population and Preventive Medicine Stony Brook University Stony Brook New York USA; ^8^ Department of Medical and Surgical Sciences University of Bologna Bologna Italy

**Keywords:** cancer prevention, cocaine inhalation, drug use, head and neck cancer

## Abstract

**Background:**

Cocaine is an illegal recreational drug used worldwide, yet little is known about whether cocaine inhalation (smoking/snorting) increases the risk of head and neck cancer (HNC).

**Methods:**

The analyses were conducted by pooling data from three case–control studies with 1639 cases and 2506 controls from the International Head and Neck Cancer Epidemiology Consortium. Epidemiologic data, including cocaine use histories, were obtained in face‐to‐face interviews. Odds ratios (ORs) and corresponding 95% confidence intervals (CIs) were estimated using hierarchical logistic regression models.

**Results:**

Controlling for cumulative tobacco and alcohol use, we observed a weak positive association between cocaine use and HNC (OR_ever vs. never_ = 1.35, 95% CI: 0.96, 1.90). In stratified analysis, while we did not detect associations among never tobacco or alcohol users due to the limited sample size, the association with cocaine use was observed among tobacco users and alcohol drinkers. ORs for ever and high cumulative use (>18 times) versus never use were 1.40 (95% CI: 0.98, 2.00) and 1.66 (95% CI: 1.03, 2.69) among tobacco users, and 1.34 (95% CI: 0.93, 1.92) and 1.59 (95% CI: 1.00, 2.51) among alcohol drinkers, respectively.

**Conclusion:**

In this pooled analysis, we observed a weak positive association between cocaine inhalation and HNC risk. Our findings provide preliminary evidence of the potential carcinogenic effect of cocaine on HNC. Because of study limitations, including limited number of cocaine users, confounding, and heterogeneity across studies, future investigations will require larger studies with more detailed information on cocaine use history.

## 1 INTRODUCTION

Head and neck cancer (HNC) refers to cancers that originate in the upper aerodigestive tract (UADT), including the oral cavity, pharynx, larynx, paranasal sinuses and nasal cavity, and salivary glands; squamous cell carcinomas of the head and neck are the most common histological subtype.^1^, ^2^ In 2020, there were an estimated 931,931 new cases of HNC and 467,125 deaths from these malignancies worldwide, representing 4.9% and 4.7% of all new cases and deaths from cancer, respectively.^3^ Tobacco smoking is causally associated with cancers of the oral cavity, pharynx, and larynx.^4^ Furthermore, according to the International Agency for Research on Cancer (IARC), there is sufficient evidence that opium smoking causes cancer of the larynx and limited evidence that opium smoking causes cancer of the pharynx.^5^

Cocaine users administer cocaine orally, intravenously, or by inhalation (i.e., snorting and smoking). When cocaine is inhaled, its route of administration shares similarities with tobacco and opium smoking. In 2021, 1.7% (4.8 million) people in the United States aged 12 years or older had past‐year cocaine use, which made cocaine the fifth most commonly used illicit drug.^6^ In addition, it has been suggested that snorting and smoking are more common as compared to other routes of administration for cocaine in the United States and European countries.^7^, ^8^, ^9^ The increasing use of cocaine coupled with the close connection between smoking behaviors and HNC raises concerns about whether cocaine use increases the risk of these malignancies. While a few epidemiological studies have investigated the associations between cocaine use and cancer,^10^, ^11^, ^12^ none of these studies have included HNC, and the potential association between cocaine use and HNC remained unexplored.

The objective of this study is to evaluate the association between cocaine inhalation and HNC risk using the pooled dataset from the International Head and Neck Cancer Epidemiology Consortium (INHANCE).

## 2 MATERIALS AND METHODS

### 2.1 Study design and population

The INHANCE consortium allowed inclusion with invasive cancer cases of the oral cavity, oropharynx, hypopharynx, oral cavity or pharynx not otherwise specified (NOS), larynx, or HNC unspecified^13^; cases with cancers of the salivary glands or nasal cavity/ear/paranasal sinuses were excluded due to relatively different etiologies.^14^ Within version 1.6 of the INHANCE dataset, cocaine use information was available from three case–control studies (Seattle,^15^ Los Angeles,^16^ and Houston^17^) comprising 1669 cases and 2521 controls. More details on each study were summarized in Table S1. Tumor site information was provided from original studies using either the International Classification of Diseases‐Oncology, Version 2 (ICD‐O‐2) or ICD 9 or 10. We excluded cases with missing information on the site of origin of their cancer (2 cases). After further excluding 43 subjects (28 cases and 15 controls) with missing information on age, sex, race/ethnicity, education, cannabis use status, and cocaine use status, there were 1639 cases and 2506 controls. Among the 1639 HNC cases, there were 503 oral cavity cancer cases, 704 oropharyngeal cancer cases, 55 hypopharyngeal cancer cases, 134 oral cavity or pharynx NOS cases, and 243 laryngeal cancer cases.

Informed consent and institutional review board approvals were obtained within the framework of the original studies.

### 2.2 Data collection on cocaine use

For each study included in the analysis, information on cocaine use was collected during the interview. While the wording of the questionnaires varied across studies, all subjects were first asked if they had ever used cocaine and/or crack cocaine. The LA and Houston studies focused on crack smoking only; participants from the Seattle study were asked to provide the types of cocaine as well as delivery methods, and from that study only cocaine inhalation (smoking/snorting) was included in the analysis. Participants who answered yes to the initial question were then asked about the details of their cocaine use. Age at starting and quitting, and frequency of use during each period were collected in the Seattle and Houston studies. Cumulative lifetime cocaine use was then calculated by summing period‐specific frequencies over time periods. In the LA study, the cumulative lifetime cocaine use was collected as a categorical variable, with four categories: 1–10 times, 11–30 times, 31–100 times, and more than 100 times. To facilitate pooling with the Seattle and Houston study data (i.e., to obtain the distribution of cumulative use across studies), midpoint values of closed intervals (e.g., 5.5 for subjects reporting having used 1–10 times) and the smallest integers of open intervals (e.g., 101 for subjects reporting having used more than 100 times) were then assigned to each subject. Finally, based on the cumulative use distribution across three studies, the median level among control subjects was then calculated to categorize cumulative cocaine use as no more than median (≤median) or more than median (>median). In the following context, we defined these two categories as low and high cumulative use, respectively.

### 2.3 Statistical methods and data analysis

We estimated odds ratios (ORs) and 95% confidence intervals (95% CIs) using hierarchical logistic regression models with study center as a random effect. In Model 1, we adjusted for age (continuous), sex, race/ethnicity (White, Black, Hispanic, Asian or Pacific Islander, and others), and education (Junior high school or less, some high school, high school graduate, technical school or some college, and college graduate or more). In Model 2, we additionally adjusted for ever tobacco use, tobacco use pack‐years (continuous), alcohol consumption drink‐years (continuous), and ever cannabis smoking status. A previous INHANCE publication reported some associations for cannabis use after controlling for tobacco and alcohol use in the INHANCE dataset; we therefore accounted for this finding by adding cannabis use in the full model.^18^ Sensitivity analyses were conducted by removing the hospital‐based Hoston study.

We fitted models for binary use status (ever vs. never) and categorized cumulative use (low cumulative use [0–18 times] and high cumulative use [>18 times] vs. never) to assess the association between cocaine use and HNC. To evaluate the dose–response association, an interval variable (coded as 0, 1, 2…) was assigned according to the categorization of cumulative cocaine use in the LA study, since the LA study only recorded cocaine use in a categorical manner.

Stratified analyses were conducted by categories of tobacco use or alcohol consumption. Potential interactions of tobacco/alcohol use on the association of cocaine use and HNC on both the multiplicative and additive scales were examined by including a product term of cocaine use and tobacco/alcohol use in the logistic regression model. We assessed the multiplicative interaction by estimating the ratio of odds ratios (ROR), essentially the exponentiated product‐term coefficient. The additive interaction was tested through estimation of the relative excess risk due to interaction (RERI); under the rare disease assumption, ORs were used to approximate risk ratios. In addition, as HNC is a heterogeneous group of diseases etiologically, we conducted subgroup analyses by cancer site; hypopharyngeal cancer was not included due to limited number of patients.

All analyses were performed using SAS version 9.4 (SAS Institute, Cary, NC); forest plots were made using R (version 4.3.1, R Foundation for Statistical Computing, Vienna, Austria).

## 3 RESULTS

The distribution of sociodemographic characteristics and confounding factors is presented in Table 1. Compared to controls, HNC cases were older and less educated. Cases were also more likely to be male (75.2% vs. 67.0%). Ever tobacco‐users were more common in the case group (78.4%) than in controls (58.6%); in addition, 33.5% of HNC cases were heavy tobacco users (>40 pack‐years), whereas that proportion was only 10.5% in the control group. Similarly, as compared to controls, ever‐drinkers (80.5% vs. 71.9%) and heavy drinkers (more than 60 alcohol drink‐years) were over‐represented among cases (37.2% vs. 14.7%). The distribution of cannabis smoking was similar: 14.3% and 15.6% of cases and controls reported cannabis use, respectively.

**TABLE 1:** Selected characteristics of head and neck cancer cases and controls in the INHANCE consortium.

	Cases		Controls	
	*n*	%^a^	*n*	%^a^
Total	1639		2506	
Study				
Seattle	392	23.9	608	24.3
Los Angeles	425	25.9	1033	41.2
Houston	822	50.2	865	34.5
Age (years)				
<40	98	6.0	220	8.8
40–44	123	7.5	255	10.2
45–49	230	14.0	386	15.4
50–54	307	18.7	555	22.2
55–59	383	23.4	559	22.3
60–64	247	15.1	289	11.5
65–69	122	7.4	138	5.5
70–74	64	3.9	58	2.3
≥75	65	4.0	46	1.8
Sex				
Female	406	24.8	828	33.0
Male	1233	75.2	1678	67.0
Race/ethnicity				
White, non‐Hispanic	1310	79.9	1920	76.6
Black, non‐Hispanic	135	8.2	184	7.3
Hispanic	131	8.0	274	10.9
Asian/Pacific Islanders, non‐Hispanic	45	2.8	78	3.1
Others	18	1.1	50	2.0
Education level				
Junior high school or less	106	6.5	101	4.0
Some high school	283	17.3	247	9.9
High school graduate	325	19.8	402	16.0
Technical school, some college	512	31.2	835	33.3
College graduate or more	413	25.2	921	36.8
Alcohol consumption drink‐years				
Never‐drinkers	320	19.5	703	28.1
>0–20	369	22.5	914	36.5
>20–30	102	6.2	196	7.8
>30–40	95	5.8	156	6.2
>40–50	70	4.3	91	3.6
>50–60	74	4.5	77	3.1
>60	609	37.2	369	14.7
Tobacco use pack‐years				
Never‐users	354	21.6	1037	41.4
>0–10	212	12.9	561	22.4
>10–20	155	9.5	261	10.4
>20–30	158	9.6	212	8.5
>30–40	211	12.9	173	6.9
>40–50	170	10.4	110	4.4
>50	379	23.1	152	6.1
Cannabis smoking				
Never	1405	85.7	2114	84.4
Ever	234	14.3	392	15.6

^a^ Percentages may not add up to 1 due to rounding.

Cocaine use reported by cases and controls for each study is shown in Table 2. The prevalence of cocaine use in the two population‐based studies (Seattle and LA) was higher than that in the hospital‐based Houston study. In the Seattle and LA studies, 8.4% and 12.7% of the cases and 8.5% and 5.5% of the controls reported cocaine use, respectively. In contrast, cocaine use was only reported in three cases (0.4%) and eight controls (0.9%) in the Houston study.

**TABLE 2:** Cocaine use among head and neck cancer cases and controls in the INHANCE consortium, by study.

	Seattle		Los Angeles		Houston	
	Cases *n* (%)^a^	Controls *n* (%)^a^	Cases *n* (%)^a^	Controls *n* (%)^a^	Cases *n* (%)^a^	Controls *n* (%)^a^
Total	392	608	425	1033	822	865
Cocaine use						
Never	359 (91.6)	556 (91.5)	371 (87.3)	976 (94.5)	819 (99.6)	857 (99.1)
Ever	33 (8.4)	52 (8.5)	54 (12.7)	57 (5.5)	3 (0.4)	8 (0.9)
0–30 times	19 (4.9)	31 (5.1)	28 (6.6)	37 (3.6)	0	2 (0.2)
30–100 times	1 (0.3)	4 (0.7)	10 (2.4)	7 (0.7)	0	0
More than 100 times	13 (3.3)	17 (2.8)	16 (3.8)	13 (1.3)	3 (0.4)	6 (0.7)

^a^ Percentages may not add up to 1 due to rounding.

Associations between cocaine inhalation and HNC are presented in Table 3. The minimally adjusted model suggested a positive association between cocaine use and HNC with a dose–response relationship (OR_ever vs. never_ = 1.58, 95% CI: 1.17, 2.13; *p* for trend = 0.027). After controlling for tobacco use, alcohol consumption, and cannabis smoking in Model 2, we observed a slight reduction in the adjusted ORs. Nevertheless, a weak positive association remained for ever versus never cocaine use (OR = 1.35, 95% CI: 0.96, 1.90). The ORs (95% CIs) for 0–30, 30–100, and more than 100 times of cocaine use were 1.26 (0.82, 1.93), 1.73 (0.69, 4.37), and 1.40 (0.82, 2.42), respectively (*p* for trend = 0.096). While little association was observed comparing low (≤median, 18 times) cumulative cocaine use and never use, high (>median) versus never use was associated with an OR of 1.52 (95% CI: 0.98, 2.35). Results from sensitivity analyses excluding the Houston study (Table S2) indicated no material changes.

**TABLE 3:** Associations (estimated ORs and 95% CIs) between cocaine use and the risk of head and neck cancer in the INHANCE consortium.

	Cases	Controls	Adjusted OR^a^ (95% CI)	
			Model 1^b^	Model 2^c^
Cocaine use				
Never	1549	2389	1 (Reference)	1 (Reference)
Ever	90	117	1.58 (1.17, 2.13)	1.35 (0.96, 1.90)
*p* for heterogeneity			0.027	0.074
Lifetime cocaine use frequency				
Never	1549	2389	1 (Reference)	1 (Reference)
≤median^d^	38	58	1.36 (0.89, 2.08)	1.19 (0.75, 1.89)
>median	52	59	1.80 (1.21, 2.67)	1.52 (0.98, 2.35)
*p* for heterogeneity			0.083	0.18
Lifetime cocaine use frequency				
Never	1549	2389	1 (Reference)	1 (Reference)
0–30 times	47	70	1.43 (0.97, 2.10)	1.26 (0.82, 1.93)
30–100 times	11	11	2.46 (1.05, 5.73)	1.73 (0.69, 4.37)
More than 100 times	32	36	1.63 (0.99, 2.69)	1.40 (0.82, 2.42)
*p* for trend			0.004	0.096
*p* for heterogeneity			0.19	0.38

^a^ Random effects model.

^b^ Adjusted for age (continuous), sex, race/ethnicity (White, Black, Hispanic, Asian or Pacific Islander, and others), and education (Junior high school or less, some high school, high school graduate, technical school or some college, and college graduate or more).

^c^ Model 1 with additional adjustment for ever tobacco use status, tobacco use pack‐years, alcohol consumption drink‐years, and ever cannabis smoking status.

^d^ Median frequency = 18 times.

**FIGURE 1:**
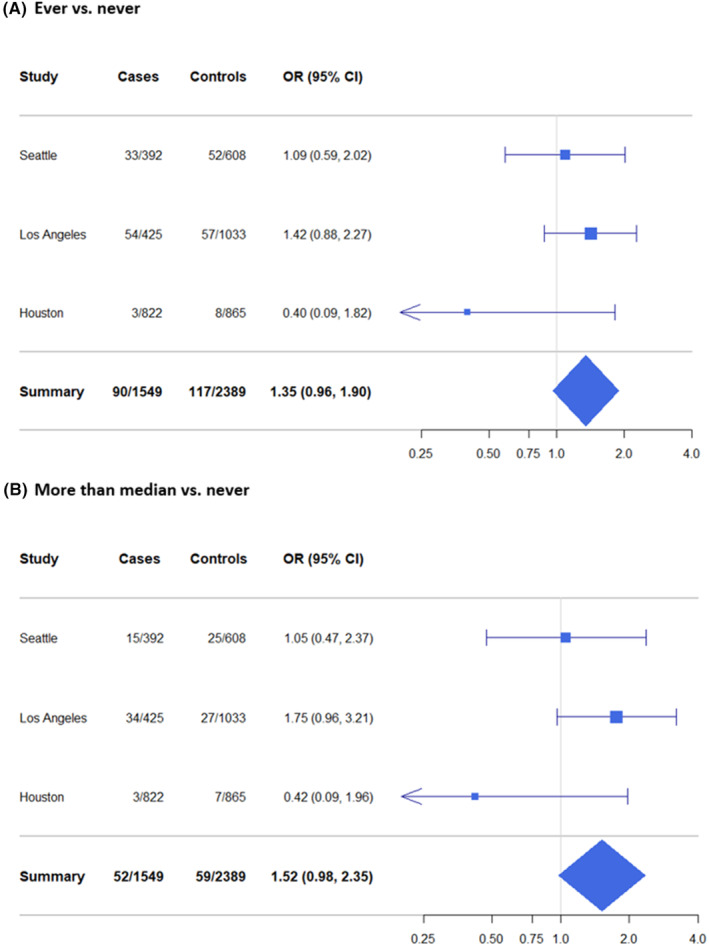
Forest plots for study‐specific associations of (A) ever versus never (reference) and (B) more than median (>18 times) versus never cocaine use with HNC among studies in the INHANCE consortium.

The forest plots (Figure 1) show the pooled and study‐specific OR estimates for the associations with HNC comparing (A) ever versus never cocaine use and (B) high versus never cocaine use. In both panels, the positive association was only seen in the LA study. We observed little or no associations in the other two studies.

Stratified analyses by never/ever tobacco use and alcohol consumption are shown in Tables 4 and 5. We repeated the main analyses among the tobacco/alcohol users, whereas only the comparison of ever versus never cocaine use was estimated among those who did not smoke or drink due to the limited number of subjects with cocaine use history. No clear associations were observed between cocaine inhalation and HNC among never tobacco users and never alcohol drinkers. The numbers of cases who were cocaine users within these two groups were small (8 and 5, respectively). In contrast, weak to moderate associations were identified among ever tobacco users (OR_ever vs. never_ = 1.40, 95% CI: 0.98, 2.00; *p* for trend = 0.092) and ever alcohol drinkers (OR_ever vs. never_ = 1.34, 95% CI: 0.93, 1.92; *p* for trend = 0.064) were similar to the association observed in the entire study population. As compared to never cocaine users, we observed increased odds of HNC among subjects with high cocaine use who had used tobacco (OR = 1.66, 95% CI: 1.03, 2.69) and had drunk alcohol (OR = 1.59, 95% CI: 1.00, 2.51). We did not detect interactions between cocaine inhalation and tobacco/alcohol use on HNC risk on any scales (Table S3).

**TABLE 4:** Associations (estimated ORs and 95% CIs) between cocaine use and the risk of head and neck cancer by tobacco use status in the INHANCE consortium.

	Never tobacco users			Ever tobacco users		
	Cases	Controls	OR^a^ (95% CI)	Cases	Controls	OR^a^ (95% CI)
Cocaine use						
Never	346	1013	1 (Reference)	1203	1376	1 (Reference)
Ever	8	24	1.08 (0.46, 2.53)	82	93	1.40 (0.98, 2.00)
*p* for heterogeneity			0.16			0.22
Lifetime cocaine use frequency						
Never				1203	1376	1 (Reference)
≤median^b^				34	48	1.20 (0.72, 1.97)
>median				48	45	1.66 (1.03, 2.69)
*p* for heterogeneity						0.25
Lifetime cocaine use frequency						
Never				1203	1376	1 (Reference)
0–30 times				42	55	1.31 (0.83, 2.09)
30–100 times				9	8	1.53 (0.54, 4.29)
More than 100 times				31	30	1.59 (0.89, 2.84)
*p* for trend						0.092
*p* for heterogeneity						0.40

^a^ Random effects model; models adjusted for age (continuous), sex, race/ethnicity (White, Black, Hispanic, Asian or Pacific Islander, and others), and education (Junior high school or less, some high school, high school graduate, technical school or some college, and college graduate or more), tobacco use pack‐years, alcohol consumption drink‐years, and ever cannabis smoking status.

^b^ Median frequency = 18 times.

**TABLE 5:** The association (estimated ORs and 95% CIs) between cocaine use and the risk of head and neck cancer by alcohol consumption status in the INHANCE consortium.

	Never alcohol drinkers			Ever alcohol drinkers		
	Cases	Controls	OR^a^ (95% CI)	Cases	Controls	OR^a^ (95% CI)
Cocaine use						
Never	315	692	1 (Reference)	1234	1697	1 (Reference)
Ever	5	11	1.17 (0.38, 3.62)	85	106	1.34 (0.93, 1.92)
*p* for heterogeneity			0.49			0.077
Lifetime cocaine use frequency						
Never				1234	1697	1 (Reference)
≤median^b^				35	54	1.15 (0.71, 1.86)
> median				50	52	1.59 (1.00, 2.51)
*p* for heterogeneity						0.062
Lifetime cocaine use frequency						
Never				1234	1697	1 (Reference)
0–30 times				43	66	1.20 (0.77, 1.88)
30–100 times				11	10	1.84 (0.72, 4.71)
More than 100 times				31	30	1.54 (0.87, 2.73)
*p* for trend						0.064
*p* for heterogeneity						0.27

^a^ Random effects model; models adjusted for age (continuous), sex, race/ethnicity (White, Black, Hispanic, Asian or Pacific Islander, and others), and education (Junior high school or less, some high school, high school graduate, technical school or some college, and college graduate or more), ever tobacco use status, tobacco use pack‐years, alcohol consumption drink‐years, and ever cannabis smoking status.

^b^ Median frequency = 18 times.

## 4 DISCUSSION

In this pooled analysis, we evaluated the association between cocaine inhalation (smoking and snorting) and HNC risk. Controlling for major confounding factors, including tobacco use and alcohol drinking, we found a weak to moderate positive association of cocaine inhalation with HNC. The association persisted when restricting the analysis to ever tobacco users and to ever alcohol drinkers.

Cocaine inhalation is associated with injuries and histopathological abnormalities in the upper airway and head and neck sites.^19^, ^20^, ^21^, ^22^, ^23^, ^24^ The carcinogenicity of cocaine was predicted previously in the early 1990s using an artificial intelligence system developed for the identification of structural determinants of biological activities.^25^ Over the past three decades, empirical studies have been conducted to assess the potential carcinogenic effect of cocaine. The genotoxicity of cocaine and the main pyrolysis product of crack cocaine has been examined in model systems. Increased DNA damage and cellular death were reported in several animal models and in vitro studies.^26^, ^27^, ^28^, ^29^ In human cells, genotoxicity was also supported by similar observations.^22^, ^30^, ^31^, ^32^ While not extensively investigated, some possible mechanisms of cocaine as a putative genotoxic substance have been proposed, including (1) cocaine‐induced oxidative stress, (2) the potential role that cocaine plays in the inflammatory process and cell cycle, and (3) genotoxic and carcinogenic potentials entailed by the complex pyrolysis products.^33^, ^34^ Additionally, recent findings of the changes in dysbiotic oral and gut microbiota resulting from cocaine use, along with the proposed association between microbiota and HNC,^35^, ^36^, ^37^, ^38^, ^39^ could provide a possibility of the connection between cocaine use and HNC. Considering these potential connections between cocaine and cancer, it would be reasonable to hypothesize the association between cocaine inhalation and HNC risk.

Studies have shown that a substantial proportion of HNC cases can be attributed to tobacco use and alcohol drinking^40^, ^41^; thus, it was crucial to consider these risk factors in our analyses. We previously reported a positive association between crack smoking and UADT cancers.^42^ However, due to different purposes of the study and corresponding methods applied in the analyses, we did not adjust for pack‐years and drink‐years, which led to a major limitation of the findings. In this study, we accounted for cumulative tobacco and alcohol use. Comparing the results from two adjusted models (Table 3), it appeared that tobacco use and alcohol drinking did not fully explain the positive association found in Model 1, and increased odds of HNC still persisted among cocaine users in the full model. However, we were unable to evaluate the associations of duration and age at start of use as these were not recorded across all studies. Given the limited assessments of the underlying association, larger studies with more detailed information on cocaine use history are needed to provide a more comprehensive evaluation.

In the stratified analysis by tobacco use and alcohol consumption, we found no evident association among the never tobacco or alcohol user groups. We did, on the other hand, observe positive associations among tobacco or alcohol users. Because of the correlation between cocaine use and tobacco/alcohol use,^43^, ^44^, ^45^ the numbers of exposed cases and controls among those who had not used tobacco and/or had not drunk were limited, and mostly consisted of light cocaine users. Even if cocaine inhalation is a true risk factor for HNC, it may be challenging to detect any effect in a small group of subjects with minimal exposure. Nonetheless, our findings among tobacco users and/or alcohol drinkers suggested that subjects among these groups who also had smoked/snorted cocaine were at a greater risk of HNC as compared to those who had not used cocaine. However, we were unable to verify whether the observed additional risk resulted from cocaine use itself or the interaction between cocaine inhalation and tobacco/alcohol use. While we did not detect such interactions, we could not rule out the possibility because the assessment was underpowered due to the limited number of exposed subjects.

Previous studies based on human oral mucosa cells indicated cocaine's potential genotoxicity.^20^, ^22^, ^31^, ^32^ Therefore, we would expect to see some associations with oral cavity cancer in the site‐specific analyses (Table S4). Nonetheless, the anticipated association with oral cavity cancer was not shown evidently, and the analyses for oropharyngeal and laryngeal cancer yielded weak associations with poor precision. Overall, the site‐specific analyses were not precise enough to be informative, likely due to the reduced sample size. Replication in a larger independent study is merited to provide a better assessment of the underlying associations and a broader interpretation of our results.

The reported prevalence of cocaine use varied considerably across studies, and we observed very few cocaine users in the Houston study. Due to this reason, when excluding the Houston study, we observed similar associations in the two population‐based studies (Table S2). Meanwhile, the prevalence of cannabis use in the Houston study was also found to be the lowest among all three studies.^46^ Whether the lower cocaine use in the Houston study data reflects greater underreporting due to more perceived stigma of admitting drug use, or a true regional difference, is unclear.

The pooled positive association was predominately driven by the LA study itself. There were a few notable differences between the Seattle and LA studies (Table S5). First, about 40% of the subjects in the LA study were non‐White, whereas the proportion was only 6% in the Seattle study. Yet, we observed similar associations when limiting the LA study to White participants (data not shown). Second, the LA study had a higher proportion of never‐tobacco‐users and never‐drinkers. Oral HPV infection is a known risk factor for oropharyngeal cancer, and many reports suggested that HPV‐related oropharyngeal cancer cases in the United States tend to have less exposure to tobacco and alcohol than other HNC cases.^47^, ^48^, ^49^ If the LA study included a substantial group of HPV‐related oropharyngeal cancer cases, the proportion of never‐tobacco‐users and never‐drinkers could be high. However, the high proportion of never‐tobacco‐users and never‐drinkers in the LA study persisted after excluding oropharyngeal cases (data not shown). Third, enrolled cases in the Seattle study seemed to be healthier than in the LA study. Eighteen percentage of eligible cases in the Seattle study died before being contacted for recruitment, while this proportion in the LA study was 10% (including the more fatal esophageal cancers, not included in this analysis).^15^, ^16^ Examining the stage at diagnosis in these two studies, we found 46% of cases in the Seattle study were diagnosed localized disease, as opposed to 32% in the LA study. The observed difference in the numbers of cases who died before being reached out could be explained if cocaine use was more extensive among HNC cases eventually diagnosed at a later stage. Fourth, the cases and controls in the Seattle study were enrolled much earlier than the LA study. Crack cocaine became popular in the United States in the 1980s, just a few years earlier than the start of the Seattle study.^43^ Among cocaine users in the Seattle study, there were only nine subjects (seven cases and two controls) who had used crack cocaine. On the contrary, the LA study only ascertained crack smoking. Because crack smoking imposes exposures to toxic pyrolysis products,^33^, ^34^ perhaps it is not surprising to see the association between cocaine use and HNC could vary across the form and route of administration of cocaine. However, because of the small number of cocaine users, we were unable to conduct informative analyses that compare cocaine use and crack use in the Seattle study.

While we attempted to include more subjects by pooling data, the pooled design is also a limitation in interpreting our findings. Given that the three studies were conducted at different times, within different populations with different sources of cases and controls and procedures for data collection, a certain degree of heterogeneity among their results was to be expected, as occurs in many pooled studies. Although test statistics did not indicate severe heterogeneity in all analyses, we acknowledge this possibility due to the small number of studies included and fit random‐effects models.

Considering the sensitivity of illicit drug use history, differential selection might have occurred in individual studies if cocaine use was associated with participation to a different extent for cases and controls. Because we were unable to assess whether there was any difference in cocaine use between participants and non‐participants among cases and controls, we could not predict the direction or magnitude of such bias. In addition, the definition of “one time of cocaine use” was imprecise, since the amount of cocaine used each time could have varied by person, time, route of administration, and the purity of cocaine. The differences in the measurement of cocaine use within each of the three studies could have led to information bias. However, such bias is likely to be non‐differential and leads to conservative estimates.

Our study could also be limited by residual confounding. As mentioned above, oral HPV infection has been suggested to be a risk factor for oropharyngeal cancer.^47^ While cocaine use has been shown to be associated with oral HPV infection in a Brazilian population,^50^ it is not clear if the association exists extensively in the US population. As we were unable to control for HPV infection in the analyses, the observed association could have partially resulted from the confounding by this factor. Nonetheless, since oropharyngeal cases comprised only a portion of our total cases, it is unlikely that the observed association was solely attributable to confounding by oral HPV. In addition, residual confounding by tobacco/alcohol use might have persisted even after controlling for the cumulative use variables of tobacco and alcohol.

In summary, the results from this pooled project support a weak‐to‐moderate positive association between cocaine inhalation and HNC that does not appear to be confounded by tobacco and/or alcohol use, or other HNC risk factors. Nevertheless, due to the small number of HNC cases that reported a history of cocaine use and other methodologic limitations, the observed association needs validation, and we are far from making causal inferences at the moment. Given the current drug use epidemic, there remains an urgent need for future work to further explore and understand the effect of cocaine use on cancer in humans.

## AUTHOR CONTRIBUTIONS

**Mingyan Zhang:** Conceptualization (equal); data curation (equal); formal analysis (equal); writing – original draft (equal); writing – review and editing (equal). **Chu Chen:** Conceptualization (equal); funding acquisition (equal); investigation (equal); writing – review and editing (equal). **Guojun Li:** Conceptualization (equal); funding acquisition (equal); investigation (equal); writing – review and editing (equal). **Alzina Koric:** Conceptualization (equal); data curation (equal); writing – review and editing (equal). **Yuan‐Chin Amy Lee:** Conceptualization (equal); data curation (equal); project administration (equal); writing – review and editing (equal). **Hal Morgenstern:** Conceptualization (equal); funding acquisition (equal); investigation (equal); writing – review and editing (equal). **Stephen Schwartz:** Conceptualization (equal); funding acquisition (equal); investigation (equal); writing – review and editing (equal). **Erich Sturgis:** Conceptualization (equal); funding acquisition (equal); investigation (equal); writing – review and editing (equal). **Paolo Boffetta:** Conceptualization (equal); project administration (equal). **Mia Hashibe:** Conceptualization (equal); data curation (equal); investigation (equal); project administration (equal); writing – review and editing (equal). **Zuo‐Feng Zhang:** Conceptualization (equal); funding acquisition (equal); investigation (equal); supervision (equal); writing – review and editing (equal).

## FUNDING INFORMATION

The Houston study was supported by the National Institutes of Health (R01ES011740 and R01CA100264). The Los Angeles Study was supported by the National Institutes of Health (Grant Numbers ES006718, ES011667, CA090388, CA077954, CA096134, DA011386, CA009142) and the Alper Research Program for Environmental Genomics of the UCLA Jonsson Comprehensive Cancer Center. The Seattle study was supported by the National Institutes of Health (R01CA048896 and R01DE012609).

## CONFLICT OF INTEREST STATEMENT

The authors declare no conflict of interest.

## ETHICS STATEMENT

Informed consent and institutional review board approvals were obtained within the framework of the original (Seattle, Los Angeles, and Houston) studies.

## Supporting information


Table S1.

## Data Availability

Data supporting the findings of our study are available via request to the International Head and Neck Cancer Epidemiology Consortium. More information can be found on the INHANCE website: https://medicine.utah.edu/dfpm/inhance.
