# Supplementary material for: Cocaine use and head and neck cancer risk: A pooled analysis in the International Head and Neck Cancer Epidemiology Consortium
Source: Cancer Med. 2024 Feb 24;13(3):e7019. doi: 10.1002/cam4.7019 (PMC10891447; doi:10.1002/cam4.7019)
Supplement: Supplementary file 1 — Table S1. [file CAM4-13-e7019-s001.docx]

**Supplement Table 1. Key characteristics of individual studies in the INHANCE consortium included in the current analysis of cocaine inhalation and HNC risk**

| **Study center** | **Recruitment period** | **Cases** | | | **Controls^a^** | |
| --- | --- | --- | --- | --- | --- | --- |
|  |  | **Source** | **Participation rate^b^** | **Age eligibility** | **Source** | **Participation rate^b^** |
| Seattle, WA | 1985-1995 | (Population-based) Cancer registry | 54%, 63% | 18-65 | Random digit dialing | 63%, 61% |
| Los Angeles, CA | 1999-2004 | (Population-based) Cancer registry | 49% | 18-65 | Neighborhood | 68% |
| Houston, TX | 2001-2006 | Hospital | 95% | >18 | Hospital visitors | 80% |

^a^All controls were frequency matched to cases on age and sex; the Los Angeles study additionally matched on neighborhood.

^b^Two responses are reported because the Seattle study data were collected in two population-based case-control studies, the first from 1985 to 1989 among men and the second from 1990 to 1995 among men and women.

**Supplement Table 2. The association (estimated ORs and 95% CIs) between cocaine use and the risk of head and neck cancer** **in the INHANCE consortium (Houston study excluded)**

|  | **Cases** | **Controls** | **Adjusted OR^a^ (95% CI)** | |
| --- | --- | --- | --- | --- |
|  |  |  | **Model 1^b^** | **Model 2^c^** |
| **Cocaine use** |  |  |  |  |
| Never | 730 | 1532 | 1 (Reference) | 1 (Reference) |
| Ever | 87 | 109 | 1.59 (1.15, 2.19) | 1.29 (0.90, 1.86) |
| P for heterogeneity |  |  | 0.073 | 0.30 |
| **Lifetime cocaine use frequency** |  | | | |
| Never | 730 | 1532 | 1 (Reference) | 1 (Reference) |
| ≤median^d^ | 38 | 57 | 1.35 (0.87, 2.11) | 1.09 (0.68, 1.77) |
| >median | 49 | 52 | 1.86 (1.21, 2.85) | 1.52 (0.95, 2.44) |
| P for heterogeneity |  |  | 0.14 | 0.43 |
| **Lifetime cocaine use frequency** |  | | | |
| Never | 730 | 1532 | 1 (Reference) | 1 (Reference) |
| 0 – 30 times | 47 | 68 | 1.40 (0.93, 2.09) | 1.18 (0.76, 1.83) |
| 30 – 100 times | 11 | 11 | 2.21 (0.93, 5.29) | 1.55 (0.61, 3.96) |
| More than 100 times | 29 | 30 | 1.81 (1.05, 3.14) | 1.46 (0.81, 2.64) |
| P for trend |  |  | 0.004 | 0.13 |
| P for heterogeneity |  |  | 0.28 | 0.65 |

^a^Random effects model.

^b^Adjusted for age (continuous), sex, race/ethnicity (White, Black, Hispanic, Asian or Pacific Islander, and others), and education (Junior high school or less, some high school, high school graduate, technical school or some college, and college graduate or more).

^c^Model 1 with additional adjustment for ever tobacco use status, tobacco use pack-years, alcohol consumption drink-years, and ever cannabis smoking status.

^d^Median frequency = 18 times.

**Supplement Table 3.** **Joint associations (estimated ORs and 95% CIs) of cocaine use and tobacco use/alcohol consumption with head and neck cancer in the INHANCE consortium**

| Tobacco use |  | | |  | | |
| --- | --- | --- | --- | --- | --- | --- |
|  | **Never tobacco users** | | | **Ever tobacco users** | | |
|  | **Cases** | **Controls** | **OR^a^ (95% CI)** | **Cases** | **Controls** | **OR^a^ (95% CI)** |
| **Cocaine use** |  |  |  |  |  |  |
| Never | 346 | 1013 | 1 (Reference) | 1203 | 1376 | 1.06 (0.86, 1.28) |
| Ever | 8 | 24 | 1.08 (0.46, 2.53) | 82 | 93 | 1.48 (1.00, 2.19) |
| ROR (95% CI) | 1.29 (0.53, 3.15) | | | | | |
| RERI (95% CI) | 0.34 (-0.67, 1.36) | | | | | |
| Alcohol consumption |  | | |  | | |
|  | **Never alcohol drinkers** | | | **Ever alcohol drinkers** | | |
|  | **Cases** | **Controls** | **OR^b^ (95% CI)** | **Cases** | **Controls** | **OR^b^ (95% CI)** |
| **Cocaine use** |  |  |  |  |  |  |
| Never | 315 | 692 | 1 (Reference) | 1234 | 1697 | 1.17 (0.97, 1.41) |
| Ever | 5 | 11 | 1.17 (0.38, 3.62) | 85 | 106 | 1.57 (1.05, 2.34) |
| ROR (95% CI) | 1.13 (0.36, 3.60) | | | | | |
| RERI (95% CI) | 0.22 (-1.18, 1.62) | | | | | |

^a^Random effects model; models adjusted for age (continuous), sex, race/ethnicity (White, Black, Hispanic, Asian or Pacific Islander, and others), and education (Junior high school or less, some high school, high school graduate, technical school or some college, and college graduate or more), tobacco use pack-years, alcohol consumption drink-years, and ever cannabis smoking status.

^b^Random effects model; models adjusted for age (continuous), sex, race/ethnicity (White, Black, Hispanic, Asian or Pacific Islander, and others), and education (Junior high school or less, some high school, high school graduate, technical school or some college, and college graduate or more), ever tobacco use status, tobacco use pack-years, alcohol consumption drink-years, and ever cannabis smoking status.

**Supplement Table 4. The association (estimated ORs and 95% CIs) between cocaine use and the risk of head and neck cancer by sites in the INHANCE consortium**

|  | **Oral cavity** | | | **Oropharynx** | | | **Larynx**^b^ | | |
| --- | --- | --- | --- | --- | --- | --- | --- | --- | --- |
|  | **Cases** | **Controls** | **OR^a^ (95% CI)** | **Cases** | **Controls** | **OR^a^ (95% CI)** | **Cases** | **Controls** | **OR^a^ (95% CI)** |
| **Cocaine use** |  |  |  |  |  |  |  |  |  |
| Never | 482 | 2389 | 1 (Reference) | 667 | 2389 | 1 (Reference) | 228 | 1833 | 1 (Reference) |
| Ever | 21 | 117 | 1.09 (0.60, 1.97) | 37 | 117 | 1.18 (0.75, 1.86) | 15 | 65 | 1.36 (0.66, 2.83) |
| P for heterogeneity |  |  | 0.90 |  |  | 0.83 |  |  | 0.23 |
| **Lifetime cocaine use frequency** |  |  |  |  |  |  |  |  |  |
| Never | 482 | 2389 | 1 (Reference) | 667 | 2389 | 1 (Reference) | 228 | 1833 | 1 (Reference) |
| ≤median (18 times) | 10 | 58 | 1.04 (0.47, 2.29) | 15 | 58 | 0.97 (0.52, 1.83) | 5 | 31 | 1.07 (0.35, 3.27) |
| >median | 11 | 59 | 1.14 (0.53, 2.47) | 22 | 59 | 1.40 (0.79, 2.48) | 10 | 34 | 1.58 (0.66, 3.81) |
| P for heterogeneity |  |  | 0.62 |  |  | 0.90 |  |  | 0.40 |
| **Lifetime cocaine use frequency** |  |  |  |  |  |  |  |  |  |
| Never | 482 | 2389 | 1 (Reference) | 667 | 2389 | 1 (Reference) | 228 | 1833 | 1 (Reference) |
| 0 – 30 times | 11 | 70 | 1.05 (0.49, 2.22) | 20 | 70 | 1.10 (0.63, 1.94) | 5 | 39 | 0.86 (0.29, 2.56) |
| 30 – 100 times | 1 | 11 | 0.65 (0.07, 5.62) | 7 | 11 | 3.13 (1.09, 9.00) | 2 | 7 | 1.47 (0.27, 8.06) |
| More than 100 times | 9 | 36 | 1.26 (0.53, 2.98) | 10 | 36 | 0.89 (0.41, 1.95) | 8 | 19 | 2.20 (0.78, 6.21) |
| P for trend |  |  | 0.68 |  |  | 0.63 |  |  | 0.17 |
| P for heterogeneity |  |  | 0.97 |  |  | 0.93 |  |  | 0.25 |

^a^Random effects model; models adjusted for age (continuous), sex, race/ethnicity (White, Black, Hispanic, Asian or Pacific Islander, and others), education (Junior high school or less, some high school, high school graduate, technical school or some college, and college graduate or more), ever tobacco use status, tobacco use pack-years, alcohol consumption drink-years, and ever cannabis smoking status.

^b^The Seattle study was excluded as there was no laryngeal cancer cases in the study.

**Supplement Table 5. Selected characteristics of head and neck cancer cases and controls in the Seattle and Los Angeles studies**

|  | **Seattle** | | **Los Angeles** | |
| --- | --- | --- | --- | --- |
|  | **Cases, n (%)*** | **Controls, n (%)*** | **Cases, n (%)*** | **Controls, n (%)*** |
| **Total** | 392 | 608 | 425 | 1033 |
| **Age (years)** |  |  |  |  |
| <40 | 27 (6.9) | 60 (9.9) | 37 (8.7) | 82 (7.9) |
| 40 - <45 | 28 (7.1) | 46 (7.6) | 35 (8.2) | 137 (13.3) |
| 45 - <50 | 53 (13.5) | 95 (15.6) | 84 (19.8) | 175 (16.9) |
| 50 - <55 | 63 (16.1) | 98 (16.1) | 110 (25.9) | 323 (31.3) |
| 55 - <60 | 81 (20.7) | 117 (19.2) | 159 (37.4) | 303 (29.3) |
| 60 - <65 | 118 (30.1) | 151 (24.8) | 0 (0) | 12 (1.2) |
| 65 | 22 (5.6) | 41 (6.7) | 0 (0) | 1 (0.1) |
| **Sex** |  |  |  |  |
| Female | 115 (29.3) | 171 (28.1) | 100 (23.5) | 416 (40.3) |
| Male | 277 (70.7) | 437 (71.9) | 325 (76.5) | 617 (59.7) |
| **Race/ethnicity** |  |  |  |  |
| White | 367 (93.6) | 572 (94.1) | 253 (59.5) | 631 (61.1) |
| Black | 15 (3.8) | 17 (2.8) | 57 (13.4) | 101 (9.8) |
| Hispanic | 0 (0) | 0 (0) | 72 (16.9) | 201 (19.5) |
| Asian/Pacific Islanders | 5 (1.3) | 7 (1.2) | 30 (7.1) | 62 (6.0) |
| Others | 5 (1.3) | 12 (2.0) | 13 (3.1) | 38 (3.7) |
| **Education level** |  |  |  |  |
| Junior high school or less | 12 (3.1) | 13 (2.1) | 43 (10.1) | 68 (6.6) |
| Some high school | 161 (41.1) | 163 (26.8) | 49 (11.5) | 46 (4.5) |
| High school graduate | 0 (0) | 0 (0) | 98 (23.1) | 184 (17.8) |
| Technical school, some college | 180 (45.9) | 334 (54.9) | 111 (26.1) | 270 (26.1) |
| College graduate or more | 39 (10.0) | 98 (16.1) | 124 (29.2) | 465 (45.0) |
| **Alcohol consumption drink-years** |  |  |  |  |
| Never-drinkers | 16 (4.1) | 45 (7.4) | 72 (16.9) | 262 (25.4) |
| >0 - 20 | 90 (23.0) | 267 (43.9) | 96 (22.6) | 422 (40.9) |
| >20 - 30 | 23 (5.9) | 66 (10.9) | 33 (7.8) | 69 (6.7) |
| >30 - 40 | 25 (6.4) | 47 (7.7) | 17 (4.0) | 64 (6.2) |
| >40 - 50 | 22 (5.6) | 29 (4.8) | 15 (3.5) | 42 (4.1) |
| >50 - 60 | 21 (5.4) | 34 (5.6) | 19 (4.5) | 26 (2.5) |
| >60 | 195 (49.7) | 120 (19.7) | 173 (40.7) | 148 (14.3) |
| **Tobacco use pack-years** |  |  |  |  |
| Never-users | 53 (13.5) | 192 (31.6) | 116 (27.3) | 471 (45.6) |
| >0 - 10 | 27 (6.9) | 130 (21.4) | 60 (14.1) | 264 (25.6) |
| >10 - 20 | 30 (7.7) | 68 (11.2) | 47 (11.1) | 99 (9.6) |
| >20 - 30 | 43 (11.0) | 73 (12.0) | 37 (8.7) | 65 (6.3) |
| >30 - 40 | 70 (17.9) | 52 (8.6) | 65 (15.3) | 73 (7.1) |
| >40 - 50 | 58 (14.8) | 33 (5.4) | 35 (8.2) | 31 (3.0) |
| >50 | 111 (28.3) | 60 (9.9) | 65 (15.3) | 30 (2.9) |
| **Cannabis smoking** |  |  |  |  |
| Never | 333 (84.9) | 516 (84.9) | 298 (70.1) | 796 (77.1) |
| Ever | 59 (15.1) | 92 (15.1) | 127 (29.9) | 237 (22.9) |
| **Stage at diagnosis** |  |  |  |  |
| Localized or earlier | 180 (45.9) |  | 136 (31.9) |  |
| Regional | 200 (51.0) |  | 201 (47.2) |  |
| Distant | 0 (0) |  | 30 (7.0) |  |
| Unstaged | 12 (3.1) |  | 59 (13.9) |  |

*Percentages may not add up to 1 due to rounding.
